# Body composition changes during breast cancer preventive treatment with anastrozole: Findings from the IBIS-II trial

**DOI:** 10.1016/j.pmedr.2024.102620

**Published:** 2024-01-22

**Authors:** Mary Pegington, Hui Zhen Tam, Adam Brentnall, Ivana Sestak, Judith Adams, Glen M. Blake, D. Gareth Evans, Anthony Howell, Jack Cuzick, Michelle Harvie

**Affiliations:** a The Prevent Breast Cancer Research Unit, The Nightingale Centre, Manchester University NHS Foundation Trust, Manchester, UK; b Division of Cancer Sciences, School of Medical Sciences, Faculty of Biology, Medicine and Health, The University of Manchester, Wilmslow Road, Manchester M20 4BX, UK; c Centre for Evaluation and Methods, Wolfson Institute of Population Health, Queen Mary University of London, UK; d Centre for Prevention, Detection and Diagnosis, Wolfson Institute of Population Health, Queen Mary University of London, UK; e Centre for Imaging Sciences, University of Manchester, Manchester, UK; f School of Biomedical Engineering and Imaging Sciences, King’s College London, St Thomas' Hospital, London, UK; g Manchester Breast Centre, Manchester Cancer Research Centre, University of Manchester, 555 Wilmslow Rd, Manchester, UK; h Division of Informatics, Imaging and Data Sciences, School of Health Sciences, Faculty of Biology, Medicine and Health, University of Manchester, Manchester, UK; i NW Genomic Laboratory Hub, Manchester Centre for Genomic Medicine, Manchester University Hospitals NHS Foundation Trust, UK; j Genomic Medicine, Division of Evolution and Genomic Sciences, The University of Manchester, St Mary’s Hospital, Manchester University NHS Foundation Trust, Oxford Road, Manchester, UK; k Department of Medical Oncology, The Christie NHS Foundation Trust, Wilmslow Rd, Manchester, UK

## Abstract

**Background:**

Uptake to anastrozole for breast cancer prevention is low, partly due to women’s concerns about side effects including gains in weight and specifically gains in body fat. Previous evidence does not link anastrozole with gains in weight, but there is a lack of data on any effects on body composition i.e. changes in fat and fat free mass. Here we assess association of anastrozole with body composition changes in a prospective sub-study from the second international breast intervention trial (IBIS-II).

**Methods:**

Participants had DXA scans at baseline and for five years of anastrozole/placebo and beyond (between March 2004 and September 2017. Primary outcomes were changes in body weight, body fat and fat free mass at 9–18 months. A linear model was used to estimate the size of a differential effect in these outcomes by randomised treatment allocation adjusted for baseline value and time since last scan, age, 10-year breast cancer risk, smoking and HRT status.

**Results:**

203 postmenopausal women were recruited (n = 95 anastrozole, n = 108 placebo), mean age 58 years (SD = 5.4), BMI 28.0 kg/m^2^ (SD = 5.5). There was no evidence of a strong association between anastrozole or placebo and endpoints at 9–18 months; effect size (95 %CI) for anastrozole minus placebo for body weight (per/kg) −0.11 (−1.29–1.08); body fat 0.11 (−0.75–0.96) and fat free mass −0.30 (−0.79–0.19).

**Conclusions:**

There is unlikely to be a clinically significant change to body composition with anastrozole for breast cancer prevention.

## 1 Introduction

### 1.1 Background

Breast cancer (BC) is the most common cancer in the UK with around 55,200 diagnoses annually (Cancer Research UK, 2021). In the UK, the National Institute for Health and Care Excellence (NICE)recommend preventive therapy to women at high risk (≥30 % lifetime risk) of breast cancer, and consideration for women at moderately increased risk (>17 % and < 30 % lifetime risk)(NICE, 2013). Current options are tamoxifen for pre- or postmenopausal women, and anastrozole or raloxifene for postmenopausal women, for five years.

Tamoxifen, a selective oestrogen-receptor modulator (SERM), reduces BC risk by ∼32 % compared to placebo and anastrozole, an aromatase inhibitor (AI), reduces risk by ∼52 % (Cuzick et al., 2014, Mocellin et al., 2019). Raloxifene, another SERM, is ∼25 % less effective than tamoxifen(Vogel et al., 2010).

Uptake to preventive therapy for breast cancer is relatively low at around 1 in 4 in trial settings and 1 in 10 in non-trial settings (Smith et al., 2016). Greater uptake is required for preventive therapy to have a greater impact on breast cancer risk reduction. There is therefore a need to understand barriers to uptake.

Patient concern over side effects from SERMS and AIs is an important issue (Flanagan et al., 2019, Jones et al., 2021, Macdonald et al., 2020, Meiser et al., 2017, Padamsee et al., 2021, Razzaboni et al., 2013, Smith et al., 2016). The main concerns relate to menopausal symptoms such as hot flushes. However, weight gain is also cited to be a concern (Jones et al., 2021). Some studies have found greater concern about side-effects is associated with lower uptake (Smith et al., 2016).

Anastrozole could theoretically alter body weight and body composition through its oestrogen lowering effects,thereby altering oestrogen’s influence on body weight, fat, and fat free mass (FFM) (Leeners et al., 2017). Change in body weight for the IBIS-II trial have already been reported (Sestak et al., 2012). After 12 months of follow-up there was little difference in weight change on anastrozole compared with placebo. Women randomised to anastrozole gained a mean (SD) 0.8 (5.3) kg compared with 0.5 (7.3) kg in the placebo group (p = 0.5). The majority of women on both anastrozole and placebo had stable weight (49.7 % and 52.4 % respectively), while 22.3 % and 21.1 % respectively lost greater than 2 kg, 18.1 % and 18.3 % respectively gained between 2 and 5 kg; and 9.9 % and 8.2 % respectively gained more than 5 kg (all p ≥ 0.4). However this overall weight measure is a crude indicator of changes in body composition, as weight can be stable alongside gains in body fat and reductions in FFM. There are few studies on the effects of anastrozole and other AIs on body composition. Those that exist are from the adjuvant setting (Nyrop et al., 2016), where changes may result from other associated treatment factors and behaviour change associated with cancer diagnosis rather than specific effects of anastrozole inhibitors.

Since patient concern about gains in weight and body fat may hinder uptake to anastrozole, it is important to assess evidence for or against this. This paper reports changes in weight and body composition within a subset of patients within the second International Breast Intervention Trial (IBIS-II), an international, randomised, double-blind, placebo-controlled trial testing anastrozole vs. placebo amongst postmenopausal women (n = 3,864) aged 40–70 years who were at increased risk of BC (registration number ISRCTN31488319) (Cuzick et al., 2014). This placebo controlled prospective prevention study in women unaffected by breast cancer provides a unique opportunity to directly assess whether anastrozole exerts effects on body composition.

### 1.2 Objectives

The aims of the IBIS-II Body Composition Sub-study were to assess the short (up to 18 months) and long-term effects (up to 66 months) of anastrozole when used to prevent BC on weight and body composition (body fat and FFM assessed using DXA) in postmenopausal women taking anastrozole compared to placebo.

## 2 Methods

### 2.1 Study design

This is a prospective cohort nested in the IBIS-II randomised control clinical trial (Cuzick et al., 2014).

### 2.2 Setting

Postmenopausal women aged 40–70 years were recruited from 153 recruitment centres in 18 countries. Between March 2004 and February 2012 women from three UK centres (Manchester, Bristol and Macclesfield) were invited to join the IBIS-II Body Composition Sub-Study at the time they were recruited to the main IBIS-II study. The sub-study required additional whole body DXA scans.

### 2.3 Participants

Eligibility and recruitment procedures for main IBIS-II Study have been detailed elsewhere (Cuzick et al., 2014). Briefly, exclusion criteria for the IBIS-II trial included premenopausal status, a diagnosis of invasive cancer in the previous five years, present use of selective oestrogen receptor modulators (SERMs) for more than six months, intention to continue with hormone replacement therapy, and evidence of severe osteoporosis. Women were excluded from the Body Composition Sub-Study if they had endocrine abnormalities, for example diabetes or hyper/hypothyroidism, if they had taken any medication known to effect body composition within 12 months before joining the study for example corticosteroids or megestrol acetate, or if they had metal implants which affect DXA body composition measurements, for example hip prostheses. High five-year adherence has already been reported in the IBIS-II trial for both anastrozole (74.6 %) and placebo (77.0 %, HR 0.89, 95 % CI 0.79–1.01, p = 0.081) (Cuzick et al., 2020).

All subjects gave informed consent. The sub-study was sponsored by University Hospital of South Manchester (now Manchester University NHS Foundation Trust, MFT) and approved by the UK North West Multicentre Research Ethics Committee MREC 02/08/70 and MREC 02/08/71. Trial registration number ISRCTN31488319.

### 2.4 Variables

The primary outcomes were changes in body weight (or BMI), body fat and FFM at 9 to <18 months follow-up since baseline across the treatment period with anastrozole and placebo. Primary interest was in comparison between randomised preventive therapy groups (anastrozole or placebo). Personnel performing the above measurements, inputting and cleaning trial data were blinded to anastrozole/placebo allocation. A statistical analysis plan was developed for this study blinded to allocation. In the analysis some adjustments used baseline covariates including age (years, continuous), 10-year BC risk (%, continuous), smoking status (ever, never, unknown), hormone replacement therapy (HRT) status (current, not current, unknown), baseline value of the outcome measure (continuous), and time since last scan (years, continuous).

### 2.5 Measurements

The sub-study protocol specified the following baseline measurements using standardised methods assessed before the first prescription of anastrozole or placebo, 1 year (12 ± 1 month), 3 years (36 ± 2 months), 5 years (0–2 months *before* stopping trial medication), and 7 years (24 ± 2 months after stopping medication):

1. Body fat (percentage of weight and kg) and FFM (kg) determined from whole body DXA scans (Hologic [Hologic Inc., Marlborough, MA] which were calibrated as described previously(Sestak et al., 2014)]
1. Android and gynoid fat (kg), android-gynoid ratio (ratio of percentages), visceral fat area (cm^2^), appendicular lean mass index (appendage lean mass/height^2^, i.e. muscle mass in the limbs) estimated by whole body DXA scans.
2. Height (baseline only) and weight (NIHR Cambridge Biomedical Research Centre, 2023)

For scans participants removed all metal objects, for example rings and clothing zips and were rescanned on the same scanner at all time points for consistency. Scanners passed daily quality control procedures. The raw data from the DXA scans was converted to measurements for analysis using Hologic APEX software version 5.6.0.5 (Hologic, Inc., Massachusetts, USA). Using the same software for all scans ensured consistency in measurements between scans undertaken at the different time points.

### 2.6 Study size

The body composition sub-study was designed based on recruitment of 80 subjects per arm. A priori this was anticipated to provide ∼90 % power if the effect size was a change of 0.4 standard deviations of the baseline levels of body fat and FFM.

### 2.7 Quantitative variables

Continuous variables were treated as such in the analysis where possible. For presentation, some a priori categories were also used (defined in statistical analysis plan): age was split as less than median age (58 years) and greater or equal to median age. BMI group was defined as underweight (<18.5 kg/m^2^), healthy weight (18.5–24.9 kg/m^2^), overweight (25–29.9 kg/m^2^), and obese (≥30.0 kg/m^2^). One outlying value of body weight (based on a priori thresholds <25.4 or >222.3 kg) was treated as missing.

### 2.8 Statistical analysis

Summary statistics presented were mean and standard deviation (SD), median and interquartile range (IQR), or frequencies (%) for categorical variables.

### 2.9 Primary analysis

The primary outcome was based on scans performed at 9 to <18 months after baseline. This period was used to maximum use of available data as there were a number of protocol deviations with missing scans and scans taking place at not specified time-points. Preliminary analysis suggested approximate normality of the change in the above measures between baseline and 9 to <18 months. The association between change in body weight, BMI, body fat and FFM at 9 to <18 month and baseline randomised allocation were assessed using a linear model for each response variable fitted by ordinary least squares, adjusted for covariates. As pre-specified in the statistical analysis plan, likelihood-ratio test was used to compute the (adjusted) p-value for randomised allocation and profile likelihood 95 % CI for the effect of anastrozole vs placebo. We used a profile likelihood 95 %CI rather than a Wald 95 %CI because it is known to be more robust (Venzon and Moolgavkar,1988).

*Secondary analysis* repeated the above, with adjustment only for baseline value of the outcome measure and time since baseline.

Adjusted analyses were also used to compare scans performed at 30 to 66 months vs baseline, and 66 months and above vs baseline, in patients with data for each comparison. A further analysis was conducted using these comparisons and the change at 9 to <18 months using women with complete data on all three time points – baseline, 9 to <18 months, and 30 to 66 months; or baseline, 30 to 66 months, and 66 months and above.

Inferential analyses of the body composition data over the full period using all women with one or more scan was based on linear mixed modelling of a random slope and intercept. Model development was blinded to treatment allocation.

### 2.10 Bias

Potential selection effects were assessed by comparing the study population with the wider IBIS-II population (total, and at three participating sites). Summary statistics on baseline characteristics by arm were used to assess risk of bias from loss of randomisation. Size of potential bias from differential missing measurements in follow-up between participants was assessed by comparing results from the main analysis with linear mixed models.

### 2.11 Missing baseline data

Baseline covariates with missing data included 10 year BC risk, smoking status, and HRT status. Simple (deterministic) imputation using the mean value were applied to the missing data.

### 2.12 Sensitivity analysis

Sensitivity analyses were conducted evaluating the missing outcome data at baseline for the primary analysis, where simple imputation using the mean value were applied to the missing data.

## 3 Results

### 3.1 Study reporting

The STROBE guidelines have been followed and the statement is in Supplementary Table 1.

### 3.2 Recruitment and withdrawal

**Fig. 1:**
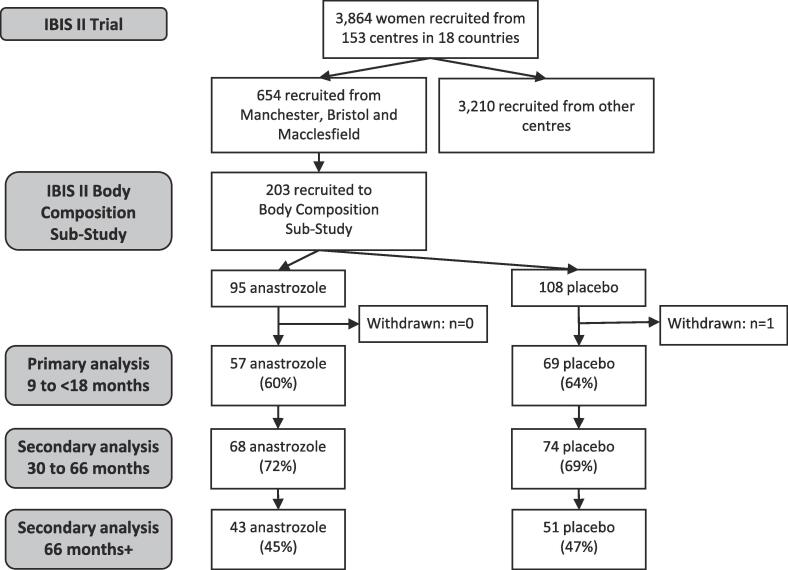
Consolidated Standards of Reporting Trials (CONSORT) flow diagram of patients recruited to the trial and during the 66 month follow up.

Of 654 participants in the main IBIS-II trial from Manchester, Bristol and Macclesfield, 203 were recruited to the body composition sub-study (95 in the anastrozole group and 108 were placebo) Sixty-two percent (n = 126) of participants had a DXA scan between nine and 18 months after baseline and were included in the primary analysis (Fig. 1). One person did not initiate therapy and shortly afterwards withdrew from the trial.

### 3.3 Baseline characteristics

The participants included in the body composition sub-study had a mean age of 58 years (SD 5.4) and BMI of 28.0 kg/m^2^ (SD 5.5) (Table 1). The majority (69 %) had a BMI above the healthy range at baseline and had never either smoked or used HRT (55 % and 64 % respectively). The characteristics of participants in the body composition sub-study were broadly similar to those of participants in main IBIS-II trial (Supplementary Table 2) and in the main trial recruited in Manchester, Bristol and Macclesfield (Supplementary Table 3). However, the proportion of women with obesity recruited to the body composition sub-study was slightly lower than that in the main trial and at the three sites of interest (27 % vs 32 % and 31 % respectively). Participants recruited from the three sites had similar characteristics to those not recruited (Supplementary Tables 4 and 5). Participants included in the primary analysis with a scan between 9 and 18 months, and in the secondary analyses with a scan 30–66 months, and 66 months and beyond had similar characteristics to those with no scan between these time points (Supplementary Tables 6-8).

**Table 1:** Baseline descriptive statistics of characteristics of adult women recruited in the placebo and anastrozole groups in the body composition sub-study conducted in the UK between March 2004 and February 2012.

Characteristic	Overall, N = 203^1^	Placebo, N = 108^1^	Anastrozole, N = 95^1^
Age (years)	58 (5.4)	57 (5.3)	59 (5.4)
<58(%)	98 (48)	56 (52)	42 (44)
≥58(%)	105 (52)	52 (48)	53 (56)
Weight (kg)	73.5 (14.7)	72.6 (14.6)	74.4 (14.9)
Unknown	4	2	2
BMI	28.0 (5.5)	27.7 (5.4)	28.4 (5.7)
Unknown	4	2	2
BMI group (%)			
<18.5	2 (1)	2 (2)	0 (0)
18.5-<25	60 (30)	33 (31)	27 (29)
25-<30	83 (42)	46 (43)	37 (40)
≥30	54 (27)	25 (24)	29 (31)
Unknown	4	2	2
Smoking (%)			
Never	107 (55)	55 (53)	52 (57)
Ever	89 (45)	49 (47)	40 (43)
Unknown	7	4	3
Breast cancer risk (10y % as per Tyrer Cuzick model v6)	7.9 (6.1, 9.9)	8.0 (6.1, 9.3)	7.8 (6.3, 10.3)
Unknown	6	4	2
HRT use ((%)			
Never user	126 (64)	64 (62)	62 (67)
Previous user	71 (36)	40 (38)	31 (33)
Unknown	6	4	2
HRT type (%)			
Oestrogen only	23 (42)	14 (45)	9 (38)
Oestrogen and progesterone	32 (58)	17 (55)	15 (62)
unknown	16	9	7
HRT duration (months)	60 (27, 108)	48 (24, 92)	72 (42, 120)

1 Mean (SD); n (%); Median (IQR).

## 4 Correlations between body measures

All body measures were correlated to each other both cross-sectionally and through time (Supplementary Tables 9-11). As expected, the strongest correlations were between body fat and both android and gynoid fat.

### 4.1 Primary analysis

There was no evidence of a strong association between anastrozole or placebo and any of the endpoints (body weight, BMI, body fat, FFM) at the primary outcome timepoint of 9–18 months (Table 2). After adjustments, the anastrozole group weighed ∼0.1 kg less on average and had ∼0.1 kg more body fat and ∼ 0.3 kg less FFM than the placebo group at this timepoint, but none were statistically significantly different (body weight 95 % CI −1.29 to 1.08, body fat −0.75 to 0.96, FFM −0.79 to 0.19). The 95 % confidence intervals indicate that there might be a change, but it is likely to be less than a kilogram therefore we deem to be unlikely to be of clinical significance.

**Table 2:** Association between anastrazole compared to placebo treatment for weight and body composition measurements at 9 to <18-month follow-up for adult women in the IBIS-2 body composition sub study from measurements. Study was conducted in the UK between March 2004 and September 2017.

Endpoint	N	Effect size* (95 %CI)	P-value
Body fat-kg	126	0.11 (−0.75 to 0.96)	0.80
FFM -kg	126	−0.30 (−0.79 to 0.19)	0.21
Body weight-kg	122	−0.11 (−1.29 to 1.08)	0.85
BMI-kg /m^2^	122	−0.05 (−0.50 to 0.40)	0.82

Note: N, Number; CI, Confidence Interval.

*Effect size is the estimated mean difference (anastrozole minus placebo) adjusted for baseline value of the outcome measure, age, time since baseline, 10 year BC risk, smoking status, HRT status.

### 4.2 Secondary analysis

**Fig. 2:**
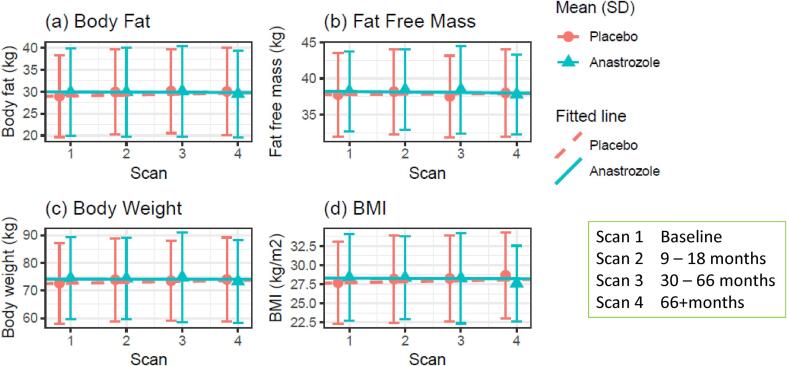
Weight, BMI and body composition measurements through the trial for adult women in the anastrazole and placebo groups of the IBIS-2 body composition sub-study The study was conducted in the UK between March 2004 and September 2017.

There were also no statistically significant differences in weight, BMI, body fat and FFM between the two groups at the different time points (Supplementary Table 12). This included the 66+ month time point which included women who had completed five years of medication or placebo. After adjustments, the anastrozole group weighed ∼0.8 kg less on average and also had ∼0.7 kg less body fat and ∼0.03 kg less FFM than placebo at 30–66 months, but all with wide confidence intervals (95 % CI −2.48 to 0.88, −1.95 to 0.55, −0.61 to 0.56 respectively). There were also no statistically significant differences in outcomes when complete cases with complete data at the different time points were included. Changes in weight, BMI, body fat and FFM were minimal over the duration of the study (Fig. 2 and Supplementary Table 13). Between baseline and 30 to 66 months, the sample mean body fat increased from 28.9 kg (SD 9.3) to 30.1 kg (9.6) in the placebo group, and 29.9 kg (9.9) to 30.1 kg (10.3) in the anastrozole group. FFM decreased from 37.8 kg (5.8) to 37.5 kg (5.6) in the placebo group and increased from 38.2 kg (5.5) to 38.5 kg (6.1) in the anastrozole group.

## 5 Discussion

### 5.1 Overall findings

We found little evidence of a differential change in weight or body composition in women randomised to receive anastrozole over five + years compared with placebo. This is important information for those potentially concerned about weight or fat gain due to taking anastrozole for BC risk reduction.

The lack of effect on weight gain with anastrozole vs placebo is consistent with the previous report by Sestak et al of no difference at 12 months which included some of the same women in the present paper (Sestak et al., 2012). The current study adds to this and shows no change in body composition measures compared with placebo in the preventive setting.

Most of the research looking at AIs and body composition studies in the adjuvant setting has not involved a placebo group so changes observed could result from the normal aging process or changes associated with a cancer diagnosis and treatments such as a reduction in physical activity. These studies have had mixed results The sample size of all these studies has been relatively small at <85 participants and most have not isolated the effect of different hormone treatments such as tamoxifen and the AIs anastrozole, letrozole or exemestane which could have different effect on body composition(Akyol et al., 2016, Battisti et al., 2014, Napoli et al., 2015). Battisti et al followed up 64 women receiving either anastrozole (n = 33) or letrozole (n = 31) after BC diagnosis and found a mean increase in abdominal visceral adipose tissue of 18.0 %, and a decrease in abdominal subcutaneous adipose tissue of 1.9 % though there was no inclusion of weight change results. Akyol et al measured body composition with bioimpedance after a year on either tamoxifen (n = 20) for premenopausal women, or letrozole (n = 35) or anastrozole (n = 22) for postmenopausal women after a diagnosis of BC. Weight showed a slight, non-significant increase from mean (SD) 73.38 (12.53) to 73.72 (13.41) kg (p = 0.69), fat mass showed a significant increase from 26.57 (8.38) to 27.61 (9.47) kg (p = 0.041), and FFM showed a non-significant decrease from 46.09 (5.96) to 45.49 (5.69) kg (p = 0.91). The changes were similar in the tamoxifen and AI groups. Napoli et al followed-up women on anastrozole (n = 62), letrozole (n = 11) and exemestane (n = 9) for one year and found non-significant changes from baseline in weight and also body composition measures: mean (SD) decrease of 1.42 (9.9) % in fat mass index, 0.77 (13.1) % in truncal fat mass index, and 0.33 (3.9) % in fat-free mass index.

Poor uptake to preventive medication is partly due to concern of side effects. Analysis of recruitment to the IBIS-II trial in Italy found that 163/319 (51 %) of women did not wish to join the trial due to concerns about side effects (Razzaboni et al., 2013). This was also the main reason for women declining preventative medication in a US high risk clinic with a documented discussion regarding chemoprevention (57/156, 36 %) (Flanagan et al., 2019). Barriers to taking preventive medication identified in a Canadian study (n = 27 recipients of, and potential candidates for preventive medication) included concern about side effects, aversion to medication, and an aversion to the term “chemoprevention” (Heisey et al., 2006). Findings of a more recent study amongst women at moderate or high risk in the UK (n = 518) supported this (Jones et al., 2021). Concern about side effects was again stated as the main barrier to taking preventive medication, with weight gain being a concern for around 20 % of participants.

There were minimal changes in weight or fat gain or reduction in lean body mass in the cohort over time. Weight gain is less in older women compared with younger women, for example a longitudinal study using English health records found a median 10 year weight gain of 5.1 kg for women in age groups between 18 and 54 years, whereas for 55–64 year olds median 10 year weight change was 1.1 kg and for 65–74 years olds it was −1.1 kg (n = 470,932) (Katsoulis et al., 2021). In the Women’s Health Initiative (n = 120,566 postmenopausal women) mean annual weight gain amongst these women with a median age of 63 years (IQR 58–69) at baseline, mean annual weight change during up to 18 years follow-up was 0.3 %, this would equate to a weight gain of 0.2 kg per year for a woman with baseline weight of 73.5 kg as in the current study. A small level of weight gain was also noted in a Finnish longitudinal study including postmenopausal women, mean 52.1 (SD 1.8) years at baseline (n = 93) (Hyvärinen et al., 2022). After mean 3.8 (SD 0.1) years follow up, weight had increased by mean 1.0 (SD 4.5) kg, fat mass by mean 1.3 (SD 4.0) kg and FFM had reduced by mean 0.5 (SD 1.5) kg.

### 5.2 Strengths, limitations and implications

We have described a unique prospective study that was designed to test the effects of anastrozole vs a placebo control group on body composition which allows comparison of the effects of anastrozole to the normal aging process. Inclusion of serial weight and standardised DXA measurements over 5 + years allowed us to study changes in body composition. A potential limitation is that the sample size for our primary endpoint of changes in body composition at 9 to <18 months was 126 rather than the planned 160 (60 % of the planned number). We accept that this may mean our analysis is under powered and could bias our results towards the null. However, the 95 % confidence intervals indicate minimal changes in body composition between the groups which are unlikely to be of clinical significance. A further limitation is that ethnicity data was not collected as part of the IBIS-II trial, so we are unable to comment on the generalisability to the UK population. The majority of participants were likely to be white. Future research should report ethnicity and assess if there are differential effects across different ethnic groups.

These analyses add to the body of evidence that anastrozole is unlikely to cause weight gain or body composition changes. This could be included in patient decision-making literature to enable a more informed choice about preventive medication.

## 6 Conclusion

We have shown in this postmenopausal, UK cohort of women at increased risk of developing BC, that anastrozole taken as a BC preventive medication does not cause significant weight change, nor significant changes to body composition measures. These data might enable women to make a more informed choice on potential harms from side effects of anastrozole to reduce their risk of breast cancer.

## CRediT authorship contribution statement

**Mary Pegington:** Formal analysis, Data curation, Writing – review & editing, Writing – original draft. **Hui Zhen Tam:** Formal analysis, Data curation, Writing – review & editing. **Adam Brentnall:** Writing – review & editing, Writing – original draft, Methodology, Formal analysis, Data curation, Conceptualization. **Ivana Sestak:** Writing – review & editing, Investigation, Data curation. **Judith Adams:** Methodology, Investigation, Conceptualization. **Glen M. Blake:** Writing – review & editing, Formal analysis, Data curation, Conceptualization. **D. Gareth Evans:** Conceptualization, Formal analysis, Methodology, Writing – review & editing. **Anthony Howell:** Writing – review & editing, Methodology, Formal analysis, Conceptualization. **Jack Cuzick:** Writing – review & editing, Methodology, Investigation, Conceptualization. **Michelle Harvie:** Writing – review & editing, Writing – original draft, Methodology, Data curation, Conceptualization.

## Declaration of competing interest

The authors declare that they have no known competing financial interests or personal relationships that could have appeared to influence the work reported in this paper.

## Data Availability

Data will be made available on request.
